# New York Odontological Society

**Published:** 1896-03

**Authors:** 

**Affiliations:** New York Odontological Society


Adjourned.

George D. B. Darby,

*Secretary.*

---

### NEW YORK ODONTOLOGICAL SOCIETY.

A regular meeting of the New York Odontological Society was held on Tuesday evening, November 19, 1895, at the New York Academy of Medicine, No. 17 West Forty-third Street, New York City, the President, Dr. Northrop, occupying the chair.

The minutes of the last meeting were read and approved.

*Dr. Jarvie*, of Brooklyn.—I have a pleasant duty to perform to-night, that of presenting to the Society a number of interesting and valuable instruments and appliances that were used in dentistry between forty and fifty years ago. They are presented to this Society by Miss Margaret Lowell Elliott, daughter of Dr. William Harvey Elliott. Dr. Elliott was born at Leicester, Mass., April 23, 1816. Studied at the Baltimore Dental College, and graduated from that institution in 1844. He began his practice at Plattsburg in this State, but in 1846 went to Montreal, where he continued to practise until 1856, when he gave up dentistry to continue his experiments and put to practical account his many inventions in fire-arms. His inventions were manufactured by the Remington Arms Company, and he continued with them until 1882, when, at the age of sixty-six, he went to Colt's armory, where he brought out his last firearm,—the lightning repeating rifle,—now being manufactured by the Colt's Company. Dr. Elliott died at Grafton, Mass., March 27, 1895. The instruments and appliances that we have here were nearly all made by Dr. Elliott himself. For ingenuity of construction and beauty of finish they cannot be excelled. In fact, although made by an amateur, I think it would put to the test the finest dental mechanism we have to-day to make instruments as fine as these are. Many of them are decidedly original, and something very similar is being put on the market to-day as new; yet this gentleman made them forty-five years ago. I think the Society is very much indebted to Miss Elliott for this disposal of many of her father's effects. I hope this will be the beginning of a large collection of such things. The evolution in dental appliances has been so rapid and has taken place in such a short space of time, that we almost forget the appliances that were in use not very long ago. I think we could get together from among the members of this Society quite a collection, and one of great interest to the student of dentistry, if each one contributed something of interest that he has in his possession.

Miss Elliott also presents to the Society her father's diploma, which was issued in 1844, and is among the first dental diplomas issued in this country; also a certificate of membership, on parchment, issued in 1842, in the American Society of Dental Surgeons. I offer the following resolutions:

WHEREAS, Miss Margaret Lowell Elliott having presented to the New York Odontological Society the two diplomas of her deceased father, Dr. William Harvey Elliott, as well as a large number of instruments and appliances used by him, and almost all of them of his own invention and made by himself; therefore,

*Resolved*, That the Society appreciate the gift as a valuable addition to its museum and as an evidence of the skill and ingenuity of one of the pioneers of dentistry in the United States.

*Resolved*, That the thanks of the Society be tendered to Miss Elliott, and a copy of this preamble and resolutions, properly engrossed and signed, be sent to her.

I am glad that Dr. John B. Rich is here to-night, for he knew Dr. Elliott and saw some of these appliances forty years ago.

*Dr. John B. Rich*, of Washington, D. C.—Mr. President, it gives me great pleasure to second that motion, for I was present when many of these beautiful instruments were exhibited to our profession by Dr. Elliott at a meeting held in New York over forty years ago.

Dr. Elliott was one of the best mechanicians I have known. These instruments were of his own invention and manufacture, and in beauty of form and perfection of finish they far excelled the production of any instrument-maker of that time. But the most remarkable feature about them was the intelligent ingenuity displayed in the construction and adaptation to the purpose for which they were intended, and in this particular they excited the most profound admiration from all who examined them. Almost every one as it was produced and its purpose explained elicited exclamations of astonishment at its ingenuity. I cannot give you any idea of the enthusiastic admiration their display excited. Dr. Edward Maynard, who was a most ingenious inventor, was paying me a visit at that time, and was present at the meeting and joined in the enthusiasm about them. After the meeting where they were exhibited adjourned, Dr. Elliott brought them to my office, and there he explained to Dr. Maynard and myself the purpose for which they had been constructed more in detail than he had been able to do before the meeting. We spent the greater part of the evening in this critical examination of these beautiful instruments, and when we had finished Dr. Maynard declared that they were the most ingenious adaptations to a purpose he had ever seen.

I recognize among these instruments one of my own invention, which Dr. Elliott made a drawing of on the evening I have mentioned. At that time artificial crowns were attached to the natural roots by means of compressed wooden dowels; when these crowns broke or became loose on the dowel, it was often very difficult to remove the dowel from the root, particularly if the periosteum was inflamed. These forceps were designed to remove the dowel without interfering with the periosteum: the protruding end of the dowel was grasped with the forceps, and the short end of the lever which is pivoted to the forceps was brought to bear upon the end of the root, and by this means the dowel was quickly removed, and without causing the roots to be forced into its socket.

I think these instruments are a great addition to the museum of this Society, and that we owe Miss Elliott our warmest thanks for presenting them to us.

INCIDENTS OF OFFICE PRACTICE.

*Dr. C. S. Stockton.*—I have had an incident of office practice,—one peculiar to myself. We all have speculated more or less concerning theory and experience. We, as dentists, no doubt have speculated and spent hours in solemn reverie concerning toothache. When a patient, with woe-begone expression and a look as if all hope had departed and the last friend gone, has presented himself, we have wondered what toothache really was and what it was like. I have had experience.

Some time since I had an approximal cavity in a lower molar filled, and the filling was contoured in such a way as to leave a pocket for the lodgement of food, the consequence being that decay increased and it was always a source of trouble and annoyance.

During the summer vacation, spent at home, an occasional gentle reminder was given that attention was required; and so I "trolleyed" to this great city of great dentists. Finally, I found one. His tone was kind, gentle, and assuring, and I found myself seated in a newly and elegantly upholstered chair. Soap and water were freely used, and finger-nails scrupulously cleansed and the hands delicately perfumed. And then the boring and the cutting began and continued; and, finally, a diagnosis made that the cavity in one tooth was dangerously near the pulp, and in the other he thought the pulp was dead. He, however, would fill and bridge with gutta-percha temporarily, and if I had any trouble his assistant would attend to me, and with a Chesterfieldian benediction I was on my way to Jersey again, toothache banished, confidence in and respect for my dentist swelling with every heart-beat.

Thus the days and the nights passed until the hot Thursday. The live pulp or the dead one had evidently felt the state of the thermometer and sympathized with it, and so ice was applied and the pulp soothed until dinner was served and the hot soup touched it, and then,—well, I thought it was too hot to eat. I used Darby's dental plasters, hot water-bags, ice-bags, pain-killers, whiskey, externally and internally, and yet the pain grew and the tooth with it. Early in the morning a neighboring dentist was sought. Soap and water were not used, and the nails were not cleansed, and the hands not perfumed. No, the time did not permit these delicate attentions, because we wanted a hole in that tooth quick, very quick.

The moral of all this is: Never have a tooth contoured so as to pocket food, and the conviction is firm that theory is better than experience, when toothache is your own.

*Dr. William B. Davenport.*—I wish to describe and exhibit a little mechanical device, which was made to be permanently attached to the lower incisors and cuspids of a patient whose teeth had become exceedingly loose from tartar and consequent disease of the gums. Certain restrictions had been put upon my efforts by the patient, as, for instance, a demand that whatever I made for him should have no clasps, that it should not annoy the tongue, and that it should not show, and I think the result which I hold here and which is a model of the piece inserted, quite interesting and helpful to others.

Having tied the six lower front teeth together with silk, an impression of them was taken with moldine and a cast obtained with Melotte's metal. A strip of pure gold thin enough to be burnished into place with hand instruments was applied to the lingual surfaces of the teeth on the cast. It covered the upper half of the teeth, curving very slightly around onto the sides of the cuspids, and not quite coming up to the cutting edges of the whole six. The cuspids were firm, but the incisors very loose, which prevented mastication to a great extent. This strip of gold, after being perfectly fitted as described, was strengthened with solder filings, and then pierced with a drill in six places to correspond with holes already made in the teeth, and large enough to admit threaded platinum pins of the size of those in plate teeth, from one-eighth to three-sixteenths of an inch long, as the thickness of the teeth would allow. All the teeth were alive, but the drilling, which was done with the right-angle hand-piece, was accomplished with comparatively no pain, as oil of cloves was used constantly and no pulps were exposed. The drill-holes were above the pulps in each instance, but as near to them as was safe. The alignment of but one pin at a time with its two drill-holes in gold strip and tooth was obtained at a sitting. A drop of wax held the pin securely to the gold, as it rested in position in its hole in the tooth and permitted the removal of the piece without bending the pin. Investing and soldering followed, and was performed six separate times. Finally, the strip of gold plate holding its six pins was set into the teeth with thin oxyphosphate of zinc, and allowed to remain thirty minutes (the dam having been previously applied) before allowing saliva to touch it. It has now been worn with comfort over two weeks, and mastication is performed without trouble.

The secretary read a communication from Philadelphia, inviting the members of the Society to celebrate the fiftieth anniversary of the organization of the first dental society in that place.

*Dr. Thomas*, of Philadelphia.—As chairman of that committee, I would say that we would be glad to have you come to meet us at that time. Philadelphia is getting to be quite noted for celebrating fiftieth anniversaries, and we would like to have as many as possible come and meet us on that occasion.

*The President.*—This appears to be a very joyous celebration of our Society, when we look around and see so many friends. Philadelphia has sent a good representation, and we have also with us to-night representatives from Washington, Boston, New Haven, and other cities. We regard it as a compliment, and we extend to all present an invitation to take part in the discussion and the deliberations of the evening as freely and as frankly as if they were in their own societies. Gentlemen, make yourselves at home. We are happy to have you with us, and shall regard you as members for the time being.

The paper of the evening, by Dr. George D. B. Darby, of Philadelphia, will now be read. It is entitled "Is Uric Acid an Important Factor in Dental Diseases?"

(For Dr. Darby's paper, see page 143.)

DISCUSSION.

*Dr. Burchard*, of Philadelphia.—It appears to me that this question of the association of certain dental disorders with the gouty diathesis has passed the stage of hypothesis; I think it has reached the stage of actual demonstration. It must be evident to every one who studies this subject and reads the literature on it that many of these cases of erosion and phagedenic pericementitis are not also affected by what is described in the text-books as "classical gout." There may be no evidences of joint-trouble or of a family history of gout, and therefore it seems that this is a question for the general pathologist to dig into again, to determine the exact mode of origin and action upon cellular structures of uric acid. I have seen myself cases of erosion where no gouty history could be elicited at all, beyond the patients suffering from a variety of disturbances beginning with affections of the alimentary canal, and in others disorders of the liver, in the lungs, in the kidneys, and in the blood-vessels. All these organs are concerned in the oxidation of the blood which supplies the tissues, or in the elimination of waste products, and so, owing to disease of any of them, there may be faulty oxidation at given points. After the pabulum has reached certain cells, there is an oxidation of the food in those cells. There are two beliefs in regard to the origin of uric acid itself: one that it represents a midway oxidation between product xanthine and urea, and gout is, therefore, attributed to the accumulation and retention of a product in insufficient oxidation. A substance urea that should be excreted by the kidneys is replaced by a substance which is not perfectly excreted by the kidneys. In regard to this question of the presence of an excess of uric acid, it has been held that the active cause of gouty manifestations was an excess and retention of uric acid (urates) in the circulating fluids. Ebstein's theory is that there is a necrosis of the tissues in an area, which area acquires an acid reaction, the uric acid, which is insoluble in an acid is deposited in that area. Within the past two weeks, Dr. George Gould (*Medical News*, November 2) calls attention to the investigation of a German pathologist, who demonstrates that the amount of uric acid formed is but a secondary factor, a local cellular debility, possibly caused primarily by a variety of intestinal troubles,—the first the underlying cause of the presence of an excess of suboxidation products. In regard to erosion being caused by an excess of uric acid, I think that Dr. Kirk's and Dr. Edwin T. Darby's cases, reported several years ago, show without doubt that there is an association between erosion and the gouty diathesis. It is recognized that the majority of cases of erosion occur in teeth that are not susceptible to the carious process. I do not say they are hard teeth, but they resist caries. It is the type of teeth found affected by pyorrhœa alveolaris. For my part, I have always regarded that kind of teeth as the product of a form of higher organization, this due primarily to the uric acid diathesis, that the teeth become progressively more organized with age, and that the presence of the excess of uric acid in the blood or circulating fluids had a causal relationship with it. In regard to Dr. Peirce's view of the exact mode of the deposits of urates, it appears to me that it contains an error; he resolves it into a question of mere physical subsidence,—that is, a fluid in a state of agitation will hold in suspension certain particles, and when the fluid is at rest these will deposit. According to Ebstein's idea, that preceding the deposits there is necrosis of an area, it seems rational to suppose that preceding the deposit of these salts on teeth there is the death of a portion of the pericementum, an acid reaction is established in the necrotic area, and the urates insoluble in acid are deposited there. As far as the dental troubles are concerned, I think this question of the dental diseases, associated with the dyscrasia, has reached the stage of actual demonstration, and we must now turn to the general pathologist to give us a better definition and conception of the intimate nature of gout, and of the diseased processes, the various disorders that begin with indigestion and end with acute gout, and the relations between them.

*Dr. L. D. Shepard.*—Like others, I came to learn rather than to instruct. Uric acid has a double interest to me, as a dentist, and as a sufferer from gout since 1889.

I have enjoyed the instructive paper and congratulate the essayist on the success of his effort.

I have recently had a case which may be of interest to relate. The family has been under my care for twenty years. The father's teeth have required a good deal of filling, but the mother's teeth were very poor, and she died some years ago from consumption. The five daughters, ranging in age now from fifteen to twenty-three years, are highly organized, nervous, and intellectual, with teeth which, previous to the days of Dr. Black, we should have called decidedly below par, and yet, thanks to a most scrupulous care of the teeth which makes them my model family for cleanliness, they have had almost no proximate decay, nearly all of their fillings having been in the sulci. On June 23, 1894, one of the young ladies, aged about twenty, came to me complaining of extreme sensitiveness of the teeth, particularly at the necks of the bicuspids and the buccal surfaces of the molars. I found a number of softened and extremely sensitive places of commencing caries near the cervical margins. Upon testing I found the saliva decidedly acid. This condition was new, and in a patient who had previously only two proximate fillings, while about all the sulci of the molars had been filled, those of the first permanent molars having been inserted at eight years of age. I advised her to use an alkaline wash, and to see the family physician to test the urine. I suspected from the conditions of the teeth and saliva, although she claimed to be in perfect health, an excess of uric acid. A few days later she reported that this was so. On her next visit, November 17, six months after, I found the saliva still acid, and then learned that her physician had given her no constitutional treatment according to my view. All he had prescribed was a change of diet, and one of the waters to drink. He is an old man, of great experience, very eminent and wise in his day, but not up to the times. About this time I filled several buccal cavities, and advised more direct treatment for uric acid. I gave her for local use a powder, adding to my regular tooth-powder tannic acid one ounce to the pound. She has been in my office since I received the notice of this meeting, and during the past summer has had efficient constitutional treatment, with the result that the sensitiveness is all gone, and the tone of the mouth changed. All she could tell me of the treatment was that she took what her new physician called Dr. Austin Flint's prescription.

I thought this case of sufficient interest to report to-night, and so looked up my records to be accurate in dates.

The essayist has quoted liberally from Haig. While his writings are fascinating, it should be stated that his views have not been universally accepted. Many of his conclusions strike me as correct from their coincidence with my own experiences. You will remember that he was a sufferer from uric acid in the form of terrible headaches, and founded much of his deductions upon personal experimentation. In accordance with the theory that uric acid is stored up during cold weather, and eliminiated by the profuse perspiration of the hot season, all of my severe attacks have occurred in the early spring, and at a season, as he says, of increased arterial tension and lowered nervous force. He gives the preference to the alkiline salts for treatment, particularly salicylate of soda. For me this medicine is a specific, and I have it always with me. My attacks yield rapidly to it, so that I can discard my crutches in a couple of days. He does not consider lithia as effective as some, claiming that, as a result of its exhibition, uric acid is stored up, and hence that the urine tests are deceptive. A very suggestive remark of his is that laboratory reactions are not reliable bases for the reactions in vital processes.

Last winter I was fascinated with the taking advertisement of the new preparation of lithia spoken of by the essayist, which is very palatable, and I submitted myself to that treatment during the winter, trying to escape my spring attack. I used it as a prophylactic, thinking I should get through the spring all right. It was followed by the worst attack I ever had. The agent of the preparation tells me I should have taken it in hot water; perhaps I did not take it in the right way; I took the right quantity just before my meals, also on retiring, but not in hot water. I have been in the habit of taking about six pints of water during the day to flush the system.

Descended as I am from particularly pious ancestors, who were leaders in the temperance reform before temperance reform was fashionable, and always having been temperate myself, I have wondered why I should have had a gouty tendency. The probable solution is the fact that to offset the growing portliness, for some years, commencing about fifteen years ago, I adopted a moderate, not extreme Banting system of feeding, increasing the consumption of beef, and decreasing that of vegetables. I have no doubt now, in reviewing the matter, that my gout is the direct result of this diet.

The essayist endorses the theory that uric acid is the cause of pyorrhœa. I was present at the first demonstration of Dr. Riggs in regard to the surgical treatment of pyorrhœa, which before his day was practically unrecognized by the profession. I have watched the course all through. I had Dr. Riggs some twenty-five years ago in Boston to treat cases there, and knew his treatment thoroughly. I must say that of all the children who have been my patients and have grown up under my hands, whose teeth have been taken care of, and proper attention given to cleanliness, I have yet to see a case of pyorrhœa alveolaris. I do not think it possible for a child who is in the hands of a competent modern dentist, and whose teeth are kept clean (which means free of any deposit which can be reached by the instruments) to have any manifestation of pyorrhœa, no matter how much uric acid that person may have in other respects. I have not spoken on this subject before, although I have been asked to do so. I am still a believer from my experience in my office that this disease proceeds mainly from the outside and not the inside. The hopeless cases I do not wish to speak of, because they are past all practical recognition of their etiology; for in dealing with etiology you must deal with the commencement of disease,—the mild cases and not the severe ones. I have seen in children, of twelve to fifteen years, what I regard as the commencement of pyorrhœa. I have removed that with a very gentle surgical operation, and by keeping the teeth clean, twenty years after there has been no manifestation of the disease more severe than the first manifestation which I had relieved. So I cannot, from my experience, accept the theory that the origin of this manifestation is uric acid.

*Dr. Rich.*—This subject of uric acid being the cause of disease in the general system has been a matter that has attracted my attention for quite a long time. For many years the matter of erosion was a mystery to the profession,—that is, its manifestation in the particular portion of the tooth where it occurred. In many cases the peculiar shape that it took, and the highly polished surface of the parts that were denuded, was a matter that was really a mystery. Nobody seems to offer any solution for that condition. Some years ago, in conversation with a friend upon this subject of erosion, of which a great many cases just at that time happened to come under my observation, he suggested that it was an acid secretion from the margin of the gums, and upon my asking him why he thought that was the reason, he said it could not come from any other cause. Upon asking him how he accounted for the highly polished surface, he did not make any satisfactory answer. About that time I collected a great many casts of erosion, many of which presented the most beautifully polished surfaces, even when examined by the microscope. In some cases the erosion extended through at least three-quarters of the tooth. This was a mysterious condition, and one of which there was no apparent solution. From the many people to whom I showed these casts, there was no satisfactory explanation. The smooth, beautifully-polished surface was against any idea of corrosion by an acid. It seemed to be impossible. At first it was attributed to the action of tooth-powders and stiff tooth-brushes, and that was the accepted theory for twenty-five or thirty years after I commenced practice; but this often occurred in my practice where there was no hard tooth-brush used, and where the people did not use the motion that would produce this condition, because my instruction to them was that they should always use the brush with a rotary motion, and never with a motion across the teeth. This peculiar rotary motion could not produce that condition. Finally, a gentleman who was badly affected, spoke to me about it. I asked him how he lived. At that time I was devoting a great deal of attention to hygiene and the physical training of the human race.

In one particular case, where erosion occurred in a lady who was one of my patients, I said to her I did not think she took enough physical exercise. I advised her to join a physical culture class, and she did so. This erosion had progressed in the surface of the teeth possibly one-eighth of the distance. She commenced as a pupil at the school, and I took an accurate cast of her teeth where they were eroded. After six months I took another cast, and there was no perceptible increase, and her general health was improved. She stayed in that school four years, and there was no recurrence of the disease at the time she went through this system of training. That lady was not wealthy enough to ride horseback, and did not have much opportunity for exercise, and besides, she was one of the persons who had to be compelled to exercise in a class to get the amount of exercise that was required to keep her in health. About a year after the school closed I noticed that this erosion had commenced again in her teeth, and it progressed until the erosion had worked half-way through the teeth. I had casts of that case taken in wax at that time. This lady's case led me to the conclusion that those cases would not occur in persons in a good state of health. My practice at that time lay almost entirely among the wealthy people, who take the least exercise of any class of citizens, and I found that in those persons who exercise the least, when this erosion once started, it was most rapid. I came to the conclusion that it was want of proper physical tone and the want of exercise that caused it. I made observations on people who were engaged in laborious occupations, and did not find any erosion. However, the immediate cause of erosion was still a mystery. Five or six years ago, the subject of uric acid was mentioned by one of my patients who was a physician. He thought that uric acid was the cause of many diseases of the human system, among them diseases of the teeth. I asked in what way, and he said he did not know particularly, and asked me to tell him some of the diseases of the teeth which we are called upon to treat. Among other things I spoke of the prevalence of Riggs's disease, as it was then called, and four or five months afterwards he said he thought the disease was the result of want of proper physical exercise. He said he should not wonder (and I

think this was almost prophetic on his part) if it were found to be an excess of uric acid in the system. He said he had a patient who was badly troubled with erosion, and who deposited large quantities of uric acid from the urine. He said he would bring that patient to me for treatment. Having been an enthusiast on that subject, I knew pretty well how to examine a man as to his physical condition, and I found that this man was a large eater and a large consumer of meat and beer, and took very little exercise. He was a stock-broker. Almost all the carbon that was generated in his system was used in his brain in his business, and this trouble was evidently caused by uric acid. Still, I could not say that this disease of the teeth—erosion—was caused by uric acid. Quite a number of cases of erosion occurred,—one of them a very peculiar case,—which went nearly three-quarters of the way through the tooth, and past that portion where the pulp-chamber was, and, upon examining it under the microscope, I found a secondary deposit of dentine on the surfaces, which were highly polished. I have those casts to-day, where the wax is as perfectly polished as if it were taken from glass.

*Dr. Louis Faugères Bishop.*—The theory of gout has been brought up in the discussion, and a great many very vague views have been touched upon. A year ago I was asked to read a paper before the medical section of the New York Academy of Medicine on gout. I went over the literature of the subject, and was very much dissatisfied; then I happened to run across the Croonian lectures of Sir William Roberts, with an account of his experiments, and his conclusions convinced me so much of their truth that for my part I have given up all the others. Those theories are so little known that I think five minutes spent on the subject will be well employed.

Sir William Roberts first began his experiments with amorphous urates, with which we are all familiar. It is rather remarkable that previous to Roberts's investigations, no one had ever determined the chemical composition of those urates. We believed that they were a mixture of urates of ammonium, calcium, sodium, etc. But decomposition had taken place in the urine. Roberts examined the urine in the fresh state. He found that urine that had been standing very soon decomposed, and a reaction took place between the acid phosphates and the alkaline phosphates, and deposits of uric acid resulted. He found that the amorphous urates were of uniform chemical consistency, and were a chemical combination known as a quadriurate, a more complicated salt than the sodium urate. It has four chemical bonds which are satisfied by hydrogen and some base. Roberts then worked out the fact that uric acid circulated in the blood normally in the form of this quadriurate, and this was the natural form in which the uric acid always circulated in the blood. It has been known from time immemorial, since the time of Scheele, that the deposits of gout are sodium biurate; that is a simpler form than the natural form of uric acid in the blood. Roberts demonstrated by scientific experiments that what occurs in gout is the change of the uric acid from the natural quadriurate to a simpler salt, the biurate, and this change takes place when any condition exists which renders the biurate less soluble. Roberts experimented very extensively upon the solubility of the sodium urate,—that is, the form in which the uric acid is deposited in the joints and probably on the teeth. We talk a great deal about uric acid, but uric acid, as such, does not exist in the blood. It cannot. The blood is an alkaline fluid and it is not possible that an acid should exist in it. I have only seen one case where the blood became acid, and that was the case of a child who had diabetes, and the child died within a very few hours. That would happen to any of us, if our blood became acid. We speak of uric acid, but it really does not exist in the blood nor in the urine when it first escapes into the kidney; but it is a product of the decomposition of urine, which may occur after it is voided. Roberts worked the solubility of sodium urate. He found that this was influenced not so much by the acidity or alkalinity of the vehicle in which it was dissolved as by the salts contained in solution. He found that the solubility of sodium biurate is as follows:

In pure water at 100° F. about one part in one thousand.

In water containing one-tenth of one per cent. of sodium bicarbonate the solubility was diminished one-half and progressively diminished as more bicarbonate was added.

The same principle was borne out by experiments with sodium chloride, sodium sulphate, sodium salicylate, and sodium phosphate.

On the other hand, the effects of different quantities of potassium chloride were very slight. Calcium salts also diminished the solubility of the sodium urate.

Therefore, we can draw the conclusion that the administration of sodium salts (common salt is sodium chloride) would have a bad effect on gout. Some physicians have told me that they demonstrated this. It would be a point worth working out, to determine whether the use of salt has any effect on the deposits around the teeth. I would recommend to your Society this work of Roberts.

As to the treatment of gout, there is really no specific treatment. The work and experiments of Haig are of extreme interest clinically, and he is a most fascinating writer; but I do not think his theoretical conclusions are quite borne out. We rely chiefly in the treatment of gout upon making the patient take large quantities of pure water without any salts at all. As you can see from Roberts's work, mineral waters are worse than Croton water, as the salts do harm, especially the sodium salts. The potassium salts do less harm. If any drug is useful, it is potassium salts, because the potassium salts, replacing the sodium salts, would increase the solubility of the sodium biurate. We rely on hygienic measures, supplying the system with a sufficient quantity of water to carry on the chemical actions easily. We have very little confidence in any one drug, but would recommend citrate of potassium as useful as any. It is more a question of regulation of life and sound common sense. But when we dismiss the treatment of gout thus summarily we must not think that it is an easy matter. It is in the treatment of these conditions that the patience, knowledge, and skill of the physician is tested. It requires little thought to stop chills with quinine, but to so manage a gouty patient that he will not suffer from gouty symptoms is a triumph of the highest medical art.

*Dr. Louise Fiske Bryson.*—It is a great privilege to be here and listen to the paper and the discussion. The reader of the paper spoke of the trouble not occurring until between the ages of thirty and forty years. My work happens to take me part of each week into an ideal school, where there are about three hundred and fifty pupils. These children are measured and weighed and carefully examined. I make measurements and physical examinations of the girls four times a year, and owing to some early training I have had I consider the condition of the mouth very important. The children's teeth are very carefully examined, and the structure of the mouth noted, and I find quite a number of children with erosion, and they do not come from the wealthy class either. The high arched palate that was called to our attention recently exists with what I have taken for erosion and with tubercular tendencies, and one instance I would call to your attention. A child seven years of age, who had the front teeth just appearing, had very suddenly an abscess of the liver, which is said in many text-books never to occur in children. It is a very serious condition. The child was not well for a whole year after, although the abscess was cured before that time. The second teeth came in riddled with imperfections, and before that child was ten years of age there were specimens of the most beautiful dentistry in that mouth. There was a case which was probably not due to entirely uric acid diathesis, because that extraordinary condition of the teeth existed at such an early age. I have observed the fact that the teeth were not decayed. I would like to ask whether it is due to uric acid or to the retention of urea.

With regard to Dr. Haig, I think Dr. Christian Herter has certainly shown that Dr. Haig has the cart before the horse very often. It occurs to me that that question of how far the retention of urea might affect the teeth would be a very interesting one to investigate. I shall be extremely indebted to any gentleman who could enlighten me as to this child. I must thank the essayist personally for his very interesting and charming paper.

*The President.*—Dr. Goddard, of California, is with us this evening, and has a few words to say before we adjourn.

*Dr. Goddard.*—In the summer of 1894 there was held in San Francisco the first dental congress of the Pacific coast. We had delegates there from all over the State, from Oregon and Washington, and the East was also slightly represented by one gentleman from Chicago, one from Philadelphia, and one from New York. Our total attendance was between two hundred and fifty and three hundred, which we considered very good, considering the scarcity of dentists on the coast. At this congress a preliminary committee of five was appointed to take charge of the matter of another congress in 1896 or 1897. This committee organized last summer, and at a recent meeting it was decided to hold a Pacific Coast Dental Congress in August, 1897, and as chairman of that committee, I wish to extend a cordial invitation to you all as members of this Society, and individually, to attend this congress in San Francisco. The American Dental Association and the Southern Dental Association will be invited to hold sessions jointly with ours, or separately, if it may seem best.

A unanimous vote of thanks was tendered to Dr. Darby for his able paper, after which, there being no further business, the meeting adjourned.

John I. Hart, D.D.S.,
*Editor New York Odontological Society.*

